# Supplementary material for: To maximize or randomize? An experimental study of probability matching in financial decision making
Source: PLoS One. 2021 Aug 26;16(8):e0252540. doi: 10.1371/journal.pone.0252540 (PMC8389367; doi:10.1371/journal.pone.0252540)
Supplement: Attachment — Submitted filename: 40629 R2R.pdf [file pone.0252540.s002.pdf]

"To maximize or randomize? An experimental study of probability matching in financial decision making"

Response to the Reviewer's Comments

Andrew W. Lo, Katherine P. Marlowe, Ruixun Zhang

March 9, 2021

We thank both the editor and the reviewer for their very helpful comments on our work—they have greatly improved the manuscript. The revised manuscript incorporates all of these comments and suggestions and point-by-point responses are provided below (editor's and reviewer's comments are reproduced in italics for convenience). We have also provided a revised manuscript with track changes.

EDITOR

Thank you for submitting your manuscript to PLOS ONE. After careful consideration, we feel that it has merit but does not fully meet PLOS ONE's publication criteria as it currently stands. Therefore, we invite you to submit a revised version of the manuscript that addresses the points raised during the review process.

The decision to request a revision comes after consultation with a qualified reviewer who raises important points about the paper that need to be addressed. While I ask you to look at and respond to each of the points, of particular importance is ensuring that the data analysis is correctly completed. Note for instance the lack of description in the text about how repeat observations from individual subjects is treated in the regression analysis. I gave the paper a read myself and have some points I would like you to address as well. Several of these points are dealing with the manner in which the paper is discussed and where confusion may arise with readers. There are also several statistical points of concern to be addressed.

We thank the editor for the thoughtful comments and we will address all of these points below.

- *P1/2: Discussion of rationality makes it sound like the factors identified that lead to differences to the things discussed in the first paragraph are "irrational", which the lines of work discussing those things do not always*

indicate. Loss aversion for instance is not necessarily "irrational" even though it goes against what could be called "traditional" theoretical models of rationality.

We thank the editor for pointing out this potential confusion and we agree wholeheartedly. We rephrased the begining of our second paragraph to be "...humans do not always behave in the same way as what these traditional economic models predict..." In fact, the main proposition from Brennan and Lo (2011) that we are testing in this paper is arguing that one such behavior (probability matching) is not necessarily "irrational", but makes perfect sense from an evolution and adaptation perspective.

- *The probability matching example of coin flips does not do what it is trying to do. I'd suggest rewording or providing a more detailed example. The intro needs a good example for those who are not familiar with the concept.*

We thank the editor for pointing out that the probability matching example is potentially unclear to readers. We have rephrased this part and provided a more detailed explanation.

- *You say "It is interesting to note that the t-statistic is higher for those subjects who reported "Yes" than for those who reported "No". This implies that subjects who believed there was a pattern were adapting to the environment of the game more strongly". Please directly test for this difference.*

We thank the editor for pointing this out. In fact, after we account for correlation in our hypothesis testing, the effective sample size decreases and we would not have enough power to test this. Therefore we have removed this claim in the paper.

- *Table 3: It is not clear how you are setting up these tests from the text. It would seem reasonable to be identifying the "f" for each participant as the probability they choose the dominant option, and thus there would be 1 data point per individual. You then would make comparisons between your four treatments. However, you say "The number of samples in the t-test equals the number of subjects multiplied by 150 stable trials." Are you saying here that you are treating each subject as 150 different independent observations (which would be inappropriate?) This is confusing and muddles the whole results section.*

We thank the editor for pointing out this potential confusion, and we have clearly done a poor job in explaining our approach and account for correlation properly. We have now revised our approach to account for correlations in each individual's decisions, and added/revised our discussion substantially to better explain how we perform these tests. In particular:

- Section 3.2, "A model for individual behavior", explains how we treat each individual decisions as Bernoulli realizations from their (unobserved) behavior. Those Bernoulli realizations are potentially correlated.

- Section 3.4, "Decision autocorrelation", specifically estimates the autocorrelation function in individual decision making.
- Section 3.5, "Probability matching", now tests the original hypothesis using a two-sample proportion test that adjusts for correlated samples. We employ an assumption of equicorrelation for individual decisions where we can compute the adjustment analytically.

With these updates, it remains statistically significant that theoretical maximizers have a higher f than theoretical randomizers. However, we lack power in testing for different slices of the subjects across the demographic, socioeconomic, and game-specific dimensions. Therefore we have removed these results.

We would also like to point out that while a hypothesis test at the subject level (estimating one f for each subject) is valid, it would be less powerful compared to the two-sample proportion tests with correlation adjustment. Therefore we keep this approach.

> - *Discussion: The conclusion is written strangely and leads to the opposite conclusions as the paper. "Specifically, individuals in environments with more balanced payoffs between the two random outcomes show a greater degree of randomized behavior, consistent with the behavioral predictions in Brennan and Lo [44]" Your paper sets up behavior in treatments 1 and 4 (That pay for correct and do not pay for incorrect, thus very unbalanced payments) are more likely to result in decision randomization than treatments 2 and 3 (where people get paid relatively more equally for correct and incorrect answers.) This is opposite of the quote from the conclusion. Why? Your regression show analysis shows that the variable "Theory Maximizer" which you say is 1 if in treatments 2 or 3, has a positive coefficient which you say is interpreted as meaning they choose the dominant option more often, which would seem to be the opposite of engaging in more randomized behavior. The conclusion continues to say that the data did not support the table 1 theory. That data did appear to support the Evolutionarily Dominate behavior theoretical outcome. Is there something I am missing or missed in this explanation?*

We thank the editor for pointing this out. There was indeed a typo that caused the confusion, and we have fixed it now and added more clarification. It should be "Specifically, individuals in environments with ***less*** balanced payoffs between the two random outcomes show a greater degree of randomized behavior, consistent with the behavioral predictions in Brennan and Lo [44]".

With regards to the second point that we appeared to say that the data did not support the table 1 theory, what we meant was that we did not find that each individual behave exactly as what the model predicted ($f^* = 0.7$ or 1.0 exactly), perhaps due to many other unobserved factors affecting individuals' real decision-making. But we indeed find evidence to support the evolutionarily-dominant behavior in the sense that theoretical randomizers randomize more than theoretical maximizers. We thank the editor for drawing our attention to this potential confusion, and we now clarified further in the Discussion.

REVIEWER #1

Summary

This study aims to experimentally test the model predictions by Brennan and Lo (2011) on probability matching. By probability matching the authors refer to a behavioural tendency to predict the outcomes of an independent randomized event to match it underlying probability distribution. In other words, participants do not pick one outcome based on maximum utility, but pick various outcomes which correspond to the underlying probability with which these outcomes occur. Their experimental design was tested on 82 participants in a behavioural laboratory setup and consisted of 200 trials of a binary choice decision. This binary choice involved guessing if an image of Brad Pitt or Angelina Jolie would appear and with every good guess participants would accrue experimental tokens. By varying the payoff structure and the probability with which one of the two images would appear, four experimental conditions were created. Based on the evolutionary dominant strategies as laid out by Brennon and Lo (2011), experimental results indeed showed a behavioural difference between theoretical maximizers and randomizers.

Major concerns

1. Aims of this paper

The goal of this paper is to experimentally test the evolutionary model by Brannan and Lo. This aim should be made much clearer than at the end of the Introduction, where it is written now. Although the authors describe this model in some detail in 2.1, the specific environmental conditions (which leads the authors to design their payoff structure) that underlies probability matching according to the authors are not well described. As this model forms the backbone of the experimental predictions, it should be described in an intuitive clear manner.

We thank the reviewer for pointing out that our discussion of the model in Brennan and Lo (2011) was not clear enough. We have expanded Section 2.1, "Evolutionary origins of probability matching", to include more details of the Brennan and Lo (2011) model, an example that illustrates the fundamental idea behind it, and how it relates to the environment we set up in this experiment.

In addition, immediately after the background paragraphs in the Introduction, we have made it explicit that "In this paper, we present the first experimental test of the evolutionary model of Brennan and Lo." Because *PLOS ONE* does not allow citations in the abstract, we believe this is the earliest possible place in the paper to state this properly.

2. Power analysis

Did the authors perform a power analysis? As many control variables are included, I wonder if there is enough power to test the numerous relationships as laid out in the results section with only 82 participants.

We thank the reviewer for pointing out the potential limitation in power. We acknowledge that our study has only 82 participants (75 after data cleaning). We are already able to find

statistically significant evidence supporting the main behavioral prediction of Brennan and Lo (2011). In the meantime, we now stress in the Discussion that our results are limited by our sample size, and when we could not find a significant relationship, a study with more sample may change that.

> *3. Separate t-tests*
>
> *It is weird to run separate t-tests for each and every socio-demographic variable. You would need to perform a correction for multiple hypothesis testing to begin with, but also I do not see the purpose of running these t-tests. I would just stick to your logistical regression. With regard to your logistical regression, how do you control for the repeated trials at the subject level, as this probably violates independence of individual data points? This is unclear and needs to be explained better.*

We thank the reviewer for pointing out the multiple testing problem for our original set of t-tests. We acknowledge that some kind of multiple testing adjustment is needed. In fact, with the new adjustment based on correlation in individual decisions, we lack power in testing for different slices of the subjects across the demographic, socioeconomic, and game-specific dimensions. Therefore we have removed these results.

We also thank the reviewer for pointing out the issue of correlated samples in logistic regression. This is closely related to the editor's comment on the issue of correlated sample in (our original) Table 3. In addition to the changes we made in response to the previous comment (Section 3.2 and 3.4), we now provide more details of how we handle correlated samples in the regression context in Section 3.6, "Individual differences".

In particular, we apply the Newey-West heteroskedasticity and autocorrelation consistent (HAC) estimator for variance of the coefficients in our logistic regression. We also stress test our conclusions with a very high truncation parameter of the HAC-consistent estimator, and our main conclusions remain valid.

Similar to the case of hypothesis testing, for regression, operating at the level of individual behavior (estimating one f for each individual) would be less powerful than our current set up. Therefore we have kept the current approach.

> *4. Discussion*
>
> *Your discussion is a summary of your results and does not relate your findings to any other study, does not discuss any limitations of your study in much detail, or addresses the relevance and practical implications of your discussion. In short, the discussion does not read as a discussion.*

We thank the reviewer for pointing out the lack of discussion on the limitations of our study and its broader implications. We have now expanded the Discussion of our paper to acknowledge the limitation, and therefore call for future work, on consistency with regards to demographic groups, to other financial and non-financial contexts, and at different magnitudes of payoff. We have also included a discussion of the broader implications of applying the evolutionary principle to individual decision making, as well as markets and societies.

Minor concerns

5. Probability matching is also a well-known term to label a technique to elicit ambiguity preferences. I would suggest you make it clear very early on what probability matching entails in your study.

This is related to the editor's comments about clarifying what probability matching means. In addition to the rephrased example, we have also added a footnote at the end of page 2 to further clarify.

6. You explain probability matching as randomizing behaviour, but also refer to randomizing and probability as two phenomena. It is unclear if these labels are synonyms or not.

We thank the reviewer for pointing out this potential confusion. When we refer to "randomizing behavior" in the paper, we meant behavior that are non-deterministic in a general sense. We have clarified this in a footnote on page 2 that "Probability matching is one specific example of randomizing behavior. In general, randomizing behavior can be any behavior that is non-deterministic."

7. What do we learn from the neuroimaging and tDCS studies regarding probability matching in your literature review? These insights feel disconnected from the rest of the literature review. Also, I think many have no clue what a tDSC study entails, so either explain better and embed within your framework, or leave it out.

We thank the reviewer for pointing out the fact that the literature on neuroimaging are not as relevant and do not motivate our paper well either. We agree with that and therefore substantially shortened the literature review and merged it into the Introduction.

8. Why did you pick such valanced images as Brad and Angelina and did not go for a neutral binary choice? Especially, since your aim is to 'use a choice between two images rather than a more financially-related tasks (such as guessing the outcome of asset-price movements) in the hopes of triggering a more primitive form of decision-making in our subjects'. (r. 425-427). What is primitive about choosing between Brad and Angelina?

We choose two images instead of financially-related tasks because we believe financially-related tasks may be more likely to trigger economically optimizing behaviors. We have added more clarification on this choice in the Discussion.

REVIEWER #2

Finally, I head some comments back from a second reviewer who recognized late in the process a conflict of interest and withdrew from the formal process. I will pass on a few thoughts for your revision from that reviewer though. I summarize here:

**p1, ln 13-14 should be rephrased as as overconfidence and loss aversion are less "behaviors" and more psychological traits*

We thank the reviewer for pointing this out and we have rephrased in the paper.

> **p8, elaborate and clarify the position that pattern seeking might be a form of randomization.*

We thank the reviewer for pointing this out and we have added a footnote on page 9 to elaborate this point.

> **p6, quote starting "the experiment we describe...for those environments." you may want to emphasize this in the introduction.*

We agree with the reviewer that this is an important point worth emphasizing in the beginning of the paper as well. We have added a paragraph to the Introduction.

JOURNAL REQUIREMENTS

> *When submitting your revision, we need you to address these additional requirements.*
>
> *1. Please ensure that your manuscript meets PLOS ONE's style requirements, including those for file naming. The PLOS ONE style templates can be found at [long URL omitted] and [long URL omitted].*

We have made several formatting edits to meet PLOS ONE's style requirements. We are happy to make further edits if required.

> *2. We note that the grant information you provided in the 'Funding Information' and 'Financial Disclosure' sections do not match.*
>
> *When you resubmit, please ensure that you provide the correct grant numbers for the awards you received for your study in the 'Funding Information' section.*

We will make the funding information consistent. Our funding is from the MIT Laboratory for Financial Engineering, and we do not have specific grant numbers associated with such funding.

> *3. We note that you have stated that you will provide repository information for your data at acceptance. Should your manuscript be accepted for publication, we will hold it until you provide the relevant accession numbers or DOIs necessary to access your data. If you wish to make changes to your Data Availability statement, please describe these changes in your cover letter and we will update your Data Availability statement to reflect the information you provide.*

We are waiting for confirmation/approvals from the MIT Committee on the Use of Humans as Experimental Subjects (COUHES) on sharing the data publicly online. We will either provide repository information once approved, or make our data sets available upon request if sharing publicly is not allowed by the policy. We will follow up and provide updates certainly before publication.
