# Supplementary material for: To maximize or randomize? An experimental study of probability matching in financial decision making
Source: PLoS One. 2021 Aug 26;16(8):e0252540. doi: 10.1371/journal.pone.0252540 (PMC8389367; doi:10.1371/journal.pone.0252540)
Supplement: Attachment — Submitted filename: To maximize or randomize.docx [file pone.0252540.s001.docx]

**To maximize or randomize? An experimental study of probability matching in financial**

**decision making**

**By Andrew W Lo, Katherine P Marlowe, Ruixun Zhang**

**Reviewed on Feb 11, 2021**

**Summary**

This study aims to experimentally test the model predictions by Brennan and Lo (2011) on probability matching. By probability matching the authors refer to a behavioural tendency to predict the outcomes of an independent randomized event to match it underlying probability distribution. In other words, participants do not pick one outcome based on maximum utility, but pick various outcomes which correspond to the underlying probability with which these outcomes occur.

Their experimental design was tested on 82 participants in a behavioural laboratory setup and consisted of 200 trials of a binary choice decision. This binary choice involved guessing if an image of Brad Pitt or Angelina Jolie would appear and with every good guess participants would accrue experimental tokens. By varying the payoff structure and the probability with which one of the two images would appear, four experimental conditions were created. Based on the evolutionary dominant strategies as laid out by Brannon and Lo (2011), experimental results indeed showed a behavioural difference between theoretical maximizers and randomizers.

**Major concerns**

1. Aims of this paper

The goal of this paper is to experimentally test the evolutionary model by Brannan and Lo. This aim should be made much clearer than at the end of the Introduction, where it is written now. Although the authors describe this model in some detail in 2.1, the specific environmental conditions (which leads the authors to design their payoff structure) that underlies probability matching according to the authors are not well described. As this model forms the backbone of the experimental predictions, it should be described in an intuitive clear manner.

2. Power analysis

Did the authors perform a power analysis? As many control variables are included, I wonder if there is enough power to test the numerous relationships as laid out in the results section with only 82 participants.

3. Separate t-tests

It is weird to run separate t-tests for each and every socio-demographic variable. You would need to perform a correction for multiple hypothesis testing to begin with, but also I do not see the purpose of running these t-tests. I would just stick to your logistical regression. With regard to your logistical regression, how do you control for the repeated trials at the subject level, as this probably violates independence of individual data points? This is unclear and needs to be explained better.

4. Discussion

Your discussion is a summary of your results and does not relate your findings to any other study, does not discuss any limitations of your study in much detail, or addresses the relevance and practical implications of your discussion. In short, the discussion does not read as a discussion.

**Minor concerns**

5. Probability matching is also a well-known term to label a technique to elicit ambiguity preferences. I would suggest you make it clear very early on what probability matching entails in your study.
6. You explain probability matching as randomizing behaviour, but also refer to randomizing and probability as two phenomena. It is unclear if these labels are synonyms or not.
7. What do we learn from the neuroimaging and tDCS studies regarding probability matching in your literature review? These insights feel disconnected from the rest of the literature review. Also, I think many have no clue what a tDSC study entails, so either explain better and embed within your framework, or leave it out.
8. Why did you pick such valanced images as Brad and Angelina and did not go for a neutral binary choice? Especially, since your aim is to ‘use a choice between two images rather than a more financially-related tasks (such as guessing the outcome of asset-price movements) in the hopes of triggering a more primitive form of decision-making in our subjects’. (r. 425-427). What is primitive about choosing between Brad and Angelina?
